# Relugolix for oral treatment of uterine leiomyomas: a dose-finding, randomized, controlled trial

**DOI:** 10.1186/s12905-021-01475-2

**Published:** 2021-10-28

**Authors:** Hiroshi Hoshiai, Yoshifumi Seki, Takeru Kusumoto, Kentarou Kudou, Masataka Tanimoto

**Affiliations:** 1 Department of Obstetrics and Gynecology, Kindai University, 377-2 Ohnohigashi, Osaka-Sayama City, Osaka 589-8511 Japan; 2 Takeda Development Center Japan, Takeda Pharmaceutical Company Limited, 1-1 Doshomachi 4-chome, Osaka, 540-8645 Japan

**Keywords:** Relugolix, Leiomyoma, Menorrhagia, Gonadotropin-releasing hormone antagonist, Randomized controlled trial, Japan, Efficacy, Safety

## Abstract

**Background:**

Uterine leiomyomas are the most common neoplasm affecting women and frequently cause heavy menstrual bleeding and pain. Gonadotropin-releasing hormone (GnRH) receptor antagonists provide fast symptom relief and show promise as a medical (non-surgical) treatment option and as a presurgical treatment to reduce leiomyoma size. The aim of this study was to evaluate the efficacy and safety of three dose levels of oral relugolix, a small molecule GnRH receptor antagonist, in Japanese women with uterine leiomyomas and heavy menstrual bleeding.

**Methods:**

This phase 2, multicenter, double-blind, parallel-group study was conducted at 36 sites in Japan in women with uterine leiomyomas and heavy menstrual bleeding, defined as a pictorial blood loss assessment chart (PBAC) score of ≥ 120 in one menstrual cycle. Patients were randomized 1:1:1:1 to relugolix 10, 20, or 40 mg, or placebo, orally once daily for 12 weeks. The primary endpoint was the proportion of patients with a total PBAC score of < 10 from week 6 to 12. A sample size of 50 patients per group was estimated to provide ≥ 95% power, based on the comparison of relugolix 40 mg with placebo using a chi-square test with a significance level of 5% (two-sided).

**Results:**

From November 2011 to September 2012, 216 patients were randomized and 214 patients (99.1%) were analyzed. The proportion (difference vs. placebo) of patients that achieved the primary endpoint in the placebo and 10-, 20-, and 40-mg relugolix groups were 0%, 20.8% (95% confidence interval [CI]: 9.3–32.3, *P* < .001), 42.6% (95% CI: 29.4–55.8, *P* < .001), and 83.3% (95% CI: 73.4–93.3, *P* < .001), respectively. Though treatment-emergent adverse events were similar between the 20- and 40-mg groups, the incidence rates were more frequent compared with the placebo group. Most of these adverse events were mild or moderate in intensity.

**Conclusions:**

Relugolix decreased menstrual blood loss in women with uterine leiomyomas in a dose–response manner, and was generally well tolerated.

**Clinical trial registration:** ClinicalTrials.gov, https://clinicaltrials.gov/ct2/show/NCT01452659, NCT01452659 (registered 17/10/2011); JAPIC Clinical Trial Information, https://www.clinicaltrials.jp, JapicCTI-111590 (registered 31/08/2011).

**Supplementary Information:**

The online version contains supplementary material available at 10.1186/s12905-021-01475-2.

## Introduction

Uterine leiomyomas occur in more than 70% of women of reproductive age in the United States [1, 2]. Furthermore, heavy menstrual bleeding and lower abdominal pain are reported in 25–50% of women with uterine leiomyomas [1, 3]. Heavy menstrual bleeding may lead to life-threatening anemia and is a primary factor that impairs the patient’s health-related quality of life (HRQL) [4–7].

Gonadal steroids, mainly estrogen and progesterone, play an important role in myoma development and growth. Gonadotropin-releasing hormone (GnRH) agonists are commonly used for presurgical treatment of uterine leiomyomas [1, 3, 8]. However, they induce a transient increase in gonadotropins and sex hormones, resulting in a clinical flare or temporary worsening of symptoms, and typically take about 3–4 weeks to achieve a therapeutic effect [1, 9]. Selective progesterone receptor modulators and GnRH receptor antagonists are also used as newer treatments for uterine leiomyomas. Elagolix, an oral GnRH receptor antagonist, decreases uterine bleeding, restores hemoglobin levels, reduces myoma and uterine volume, and improves HRQL in patients [10]. Relugolix is another orally active small molecule GnRH receptor antagonist [11] that demonstrated approximately dose-dependent decreases in blood estradiol and progesterone levels within 3 days in healthy premenopausal women in phase 1 studies (unpublished data). In phase 3 studies, relugolix 40 mg decreased pain associated with uterine leiomyomas [12], reduced myoma and uterine volumes [13], increased hemoglobin levels [13], and was noninferior to leuprorelin injections in reducing heavy menstrual bleeding [13].

This placebo-controlled phase 2 study was designed to evaluate the efficacy and safety of relugolix once daily for 12 weeks at three dose levels in women with heavy menstrual bleeding associated with uterine leiomyomas. We hypothesized that relugolix would clinically ameliorate heavy menstrual bleeding with an acceptable tolerability profile.

## Materials and methods

### Study design

This phase 2, multicenter, randomized, double-blind, parallel-group, placebo-controlled study was conducted at 36 sites in Japan. The study was conducted in accordance with the principles outlined in the Declaration of Helsinki, the International Council for Harmonisation Guideline for Good Clinical Practice, and all applicable local regulatory requirements. The study, including protocol and informed consent form, was approved by the Institutional Review Board at each study site. Each patient provided informed consent before undergoing any study-related procedures.

**Fig. 1:**
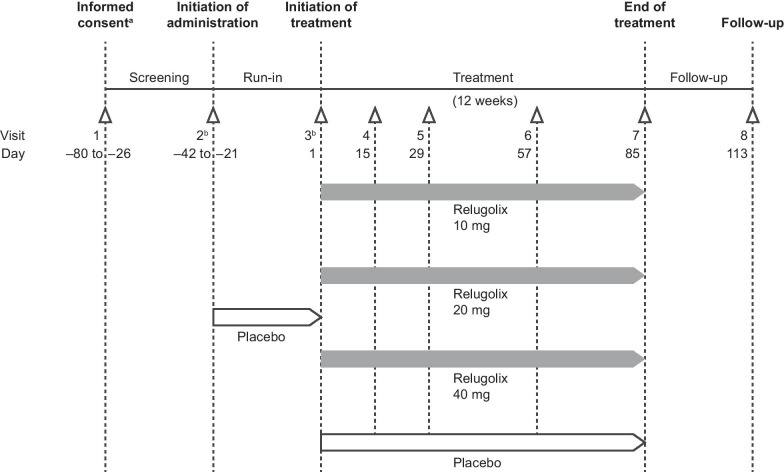
Study design. ^a^ Informed consent was obtained before day − 80. ^b^ Visits 2 and 3 occurred on days 1–5 of the menstrual cycle

The study comprised four periods: a screening period, a 3–6-week placebo run-in period, a 12-week treatment period, and a 4-week follow-up period (Fig. 1). Patients who gave informed consent at Visit 1 were screened. After screening, eligible patients entered the single-blind placebo run-in phase on days 1–5 of their first menstruation (Visit 2) and received two placebo tablets orally, 30 min before breakfast, once daily for 3–6 weeks, depending on individual cycle duration. During this period, baseline values were obtained. At the end of the placebo run-in phase (days 1–5 of the second menstruation, Visit 3), patients were randomly assigned 1:1:1:1 to one of the placebo, relugolix 10-, 20-, and 40-mg groups, in which patients received a combination of two indistinguishable tablets containing placebo, relugolix 10 or 20 mg orally, 30 min before breakfast, once daily for 12 weeks. The study drugs were supplied by Takeda Pharmaceutical Company Limited. During the treatment period, patients revisited for clinical examinations at weeks 2 (Visit 4), 4 (Visit 5), 8 (Visit 6), and 12 (Visit 7), followed by the final study visit at 16 weeks (Visit 8) (Additional file 1: Table S1).

### Patients

Eligible patients were Japanese premenopausal women who were at least 20 years of age with a diagnosis of uterine leiomyoma confirmed by transvaginal ultrasound scan, abdominal ultrasound scan, magnetic resonance imaging, computed tomography scan, or laparoscopy, and one or more measurable noncalcified myomas with a widest diameter of ≥ 3 cm. Eligible patients had regular menstrual cycles (25–38 days) and heavy menstrual bleeding in one menstrual cycle with a total pictorial blood loss assessment chart (PBAC) score of at least 120 [14, 15]. Patients were not allowed to participate if they had blood disorders (other than iron-deficiency anemia); lower abdominal pain due to irritable bowel syndrome or severe interstitial cystitis; thyroid dysfunction; pelvic inflammatory disease; a positive Pap smear test result; or a history of hysterectomy or bilateral oophorectomy, or a history of other medical conditions that could interfere with participation in the study or the evaluation of the efficacy and safety of relugolix. Inclusion and exclusion criteria are tabulated in Additional file 1: Table S2. Administration of sex hormone preparations during the study was prohibited. Oral administration of iron was allowed if the dose and regimen remained unchanged during the treatment period in patients whose blood hemoglobin level was less than 10.0 g/dL on entering the study, or patients had been using it before entering the study. Indiscriminate or prophylactical use of analgesic agents was not allowed over the study period. However, loxoprofen could be used at the discretion of the investigator to treat significant pain associated with leiomyomas, and patients recorded their pain symptoms and analgesic use (yes/no) in the patient diary before taking analgesic agents.

Eligible patients were registered by the investigator or designee via a fax communication to the participant registration center, given a sequential identification number, and allocated by the permuted block randomization method (block size: eight). The allocation table was generated by a contract research organization, Bell Medical Solutions, Inc. (Tokyo, Japan). The investigator or designee received a unique number designating a study drug set for each allocated patient. Randomization information was stored in a secured area, which was accessible only to authorized personnel. Patients and study personnel at each study site were blinded to study drug treatment assignment. Results of pharmacodynamic tests were concealed to preserve the blinding of the study until the randomization code was opened.

### Study endpoints

The study’s primary endpoint was the proportion of patients with a total PBAC score of < 10 for weeks 6–12. Twelve weeks reflect the usual presurgical treatment period for uterine leiomyomas [16]. Secondary efficacy endpoints included the proportion of patients with a total PBAC score of < 10 for weeks 2–6 and weeks 2–12; amenorrhea as assessed by the proportion of patients with a total PBAC score of 0 for weeks 6–12, for weeks 2–6, and for weeks 2–12; change in the total PBAC score relative to baseline for weeks 6–12; myoma and uterine volumes at each study visit at weeks 2–12; concentration of blood hemoglobin for visits at weeks 4–12; pain symptoms assessed daily by the numerical rating scale (NRS) score, patient-reported daily pain assessment with an 11-point scale ranging from 0 (“no pain”) to 10 (“pain as bad as you can imagine”); and the Uterine Fibroid Symptom and HRQL Questionnaire (UFS-QOL) score at each study visit at weeks 4–12 [17]. Pharmacodynamic effect-related endpoints included blood concentrations of estradiol, luteinizing hormone (LH), follicle-stimulating hormone (FSH), and progesterone at all visits. Safety was assessed by treatment-emergent adverse events (TEAEs) at every visit, vital signs, weight, 12-lead electrocardiogram (ECG), and clinical laboratory tests at each study visit at weeks 4–12, and bone mineral density (BMD) in the lumbar spine (L2–L4) assessed using dual energy X-ray absorptiometry at week 12 (Additional file 1: Table S1).

The volume of menstrual blood loss was measured using the PBAC score, which is a validated, semi-objective method of evaluating menstrual blood loss [14, 15]. Patients were required to use menstrual products designated by the sponsor and to record in a patient diary the number of tampons or pads used, clots, and flooding occurrences during the screening, run-in, and treatment periods. Transvaginal ultrasound scan was performed to determine uterine and myoma volume using the formula: D1 × D2 × D3 × π/6, where D1, D2, and D3 represent the maximal longitudinal, anteroposterior, and transverse diameter, respectively. A central laboratory (SRL Medisearch Inc., Osaka, Japan) performed laboratory tests for serum chemistry, hematology, urinalysis, and pharmacodynamics. Urine pregnancy tests were performed at the respective investigational sites.

### Statistical analysis

The sample size of this study was calculated based on a prior phase 1 study and the clinical study results of leuprolide acetate (unpublished data), which indicated that a total PBAC score of < 10 for weeks 6–12 would be achieved by 70% of patients receiving relugolix 40 mg and 10% of those receiving placebo. When a chi-square test with a significance level of 5% (two-sided) was used for comparison between the relugolix 40-mg and placebo groups, a sample size of 50 patients per group would provide ≥ 95% power. Assuming that a total PBAC score of < 10 for weeks 6–12 would be achieved by 40% of patients receiving relugolix 10 mg and 20 mg, this sample size would provide ≥ 85% overall power to show the efficacy for all comparisons between each relugolix group and the placebo group. Assuming that approximately 10% of patients would be excluded from the analysis of the primary endpoint, 55 patients had to be randomized to each group for a total sample size of 220 patients.

Efficacy was assessed in the full analysis set defined as all randomized patients who received at least one dose of study drug. Safety analyses were performed using a safety analysis set, which comprised any patient who received at least one dose of the study drug. For the primary endpoint analysis, the proportion of patients with a total PBAC score of < 10 for weeks 6–12 was summarized by treatment group. The point estimate and two-sided 95% confidence interval (CI) of the difference between each relugolix group and the placebo group were calculated. A chi-square test with a significance level of 5% (two-sided) was used for comparison between the relugolix and placebo groups. To control the type 1 error rate with multiple comparisons, between-group comparisons were performed using a closed (sequential) testing procedure, so if relugolix 40 mg was found to be significantly superior to placebo, then relugolix 20 mg versus placebo would be tested, and if relugolix 20 mg was found to be superior, then relugolix 10 mg would be compared with placebo. In addition, the primary endpoint was subjected to a subgroup analysis to examine the effect of total PBAC score at baseline.

The secondary endpoints of decrease in menstrual blood loss and amenorrhea were analyzed using a similar methodology to that used for the primary endpoint analysis. For the other secondary endpoints, summary statistics were provided by treatment group for each visit. Because not all patients had high NRS scores at baseline, change in pain was assessed in post hoc analyses in the subgroup of patients reporting a maximum NRS score ≥ 4 at baseline. Comparison between treatment groups for this subgroup analysis was performed using Fisher’s exact test.

Medical Dictionary for Regulatory Activities version 15.1 was used to code TEAEs. For continuous variables, including BMD, vital signs, weight, clinical laboratory tests, and ECGs, baseline values, observed values, and changes from baseline were summarized for each measurement time point. Missing data were not imputed. All the statistical analyses were performed using SAS version 9.2 (SAS Institute Inc., Cary, NC).

## Results

### Demographic and baseline patient characteristics

**Fig. 2:**
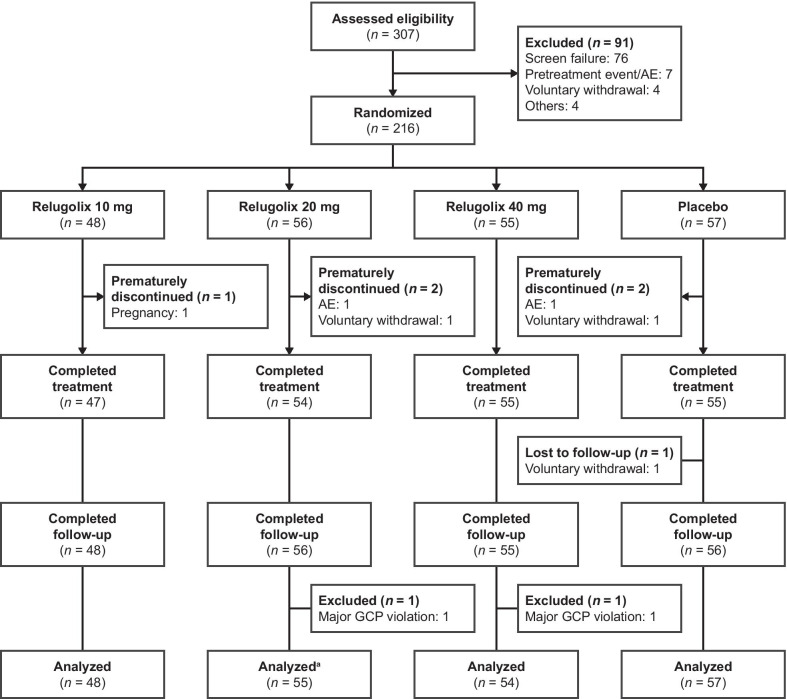
Patient disposition chart showing study participation. Of randomized patients, 214 (99.1%) were included in the full analysis and safety analysis sets. ^a^ Data for the primary endpoint were missing (*n* = 1). *AE* adverse event

From November 2011 to May 2012, 307 premenopausal women with a confirmed diagnosis of uterine leiomyoma were screened for eligibility; of these patients, 216 were randomized (Fig. 2). Five patients who received the study drug discontinued the study during the treatment period, and one did not attend the final visit during the follow-up period. Of the randomized patients, 214 (99.1%) were included in the full analysis and safety analysis sets.

The mean age of randomized patients ranged from 41 to 43 years across the treatment groups (Table 1). There were no apparent differences among the treatment groups for uterine or myoma volumes. Baseline PBAC scores were slightly higher in the placebo group compared with relugolix 10-, 20-, and 40-mg groups, although the median (quarter [Q]1, Q3) values were similar among treatment groups. No clinically significant differences for other demographic and baseline characteristics were observed among the treatment groups.

**Table 1:** Demographics and baseline characteristics

Variables	Relugolix			Placebo
	10 mg (*n* = 48)	20 mg (*n* = 56)	40 mg (*n* = 55)	(*n* = 57)
Age (years), mean ± SD	42.7 ± 4.6	42.6 ± 5.3	41.1 ± 4.4	42.4 ± 5.1
BMI (kg/m^2^), mean ± SD	22.9 ± 2.7	21.9 ± 2.8	22.4 ± 2.8	23.8 ± 4.2
Parity ≥ 1, *n* (%)	25 (52.1)	29 (51.8)	20 (36.4)	30 (52.6)
Type of uterine leiomyoma, *n* (%)				
Subserosal	22 (45.8)	25 (44.6)	17 (30.9)	23 (40.4)
Intramural	39 (81.3)	44 (78.6)	45 (81.8)	42 (73.7)
Submucosal	11 (22.9)	11 (19.6)	11 (20.0)	12 (21.1)
Cervical	1 (2.1)	1 (1.8)	2 (3.6)	1 (1.8)
Volume of myoma (cm^3^)				
Mean ± SD	115.6 ± 127.4	118.7 ± 117.4	138.0 ± 199.8	136.1 ± 159.1
Median (Q1, Q3)	61.6 (30.7, 170.9)	72.1 (27.1, 173.6)	68.2 (27.2, 167.0)	82.0 (43.6, 141.3)
Volume of uterus (cm^3^)				
Mean ± SD	322.1 ± 285.0	363.3 ± 304.6	406.6 ± 361.8	366.5 ± 276.6
Median (Q1, Q3)	212.0 (161.1, 383.8)	271.7 (172.7, 427.5)	291.0 (145.0, 557.6)	263.0 (157.0, 482.6)
PBAC score				
Mean ± SD	269.4 ± 160.8	276.5 ± 165.9	259.9 ± 190.5	327.9 ± 292.1
Median (Q1, Q3)	211.0 (139.0, 364.5)	214.0 (155.0, 331.0)	219.0 (159.0, 302.0)	232.0 (165.0, 351.0)
Pain NRS score^a^, mean ± SD	0.7 ± 1.1	0.8 ± 0.9	0.6 ± 0.6	0.8 ± 0.8
UFS-QOL score^b^, mean ± SD				
Symptom severity	29.3 ± 17.3	25.8 ± 14.4	25.3 ± 14.0	27.6 ± 17.7
HRQL total	85.7 ± 11.9	87.2 ± 11.5	85.0 ± 15.5	83.9 ± 18.8
Hemoglobin (g/dL), mean ± SD	12.2 ± 1.2	12.2 ± 1.4	12.0 ± 1.7	12.1 ± 1.5

*BMI* body mass index, *HRQL* health-related quality of life, *NRS* numerical rating scale, *PBAC* pictorial blood loss assessment chart, *UFS-QOL* uterine fibroid symptom and quality of life

^a^NRS is a patient-reported pain assessment with an 11-point scale ranging from 0 to 10. Higher NRS scores indicate worse pain

^b^UFS-QOL is a patient-reported tool to assess quality of life in patients with uterine leiomyomas. The score ranges from 0 to 100. Higher symptom severity scores indicate more severe symptoms, but higher HRQL scores indicate better HRQL

### Primary efficacy endpoint

**Fig. 3:**
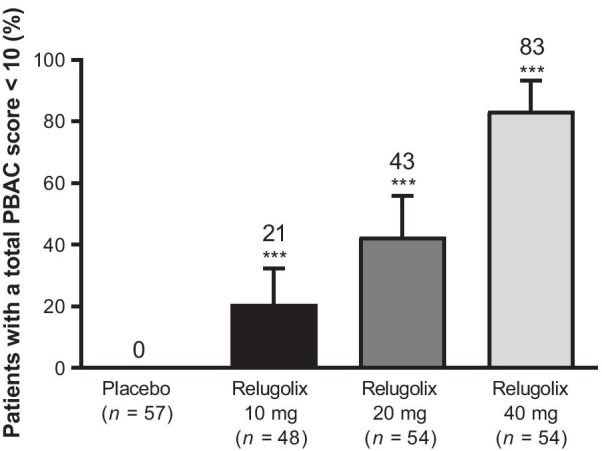
Primary efficacy endpoint: proportion of patients with a total pictorial blood loss assessment chart (PBAC) score < 10 at weeks 6–12. Error bars show 95% confidence intervals for differences vs. placebo. ***P < .001. Note: Differences were analyzed between each relugolix group and placebo group using chi-square tests according to the closed testing procedure, initially from the relugolix 40-mg group vs. the placebo group, to control the type I error rates in multiple comparison

The study met its primary endpoint: all three relugolix doses significantly reduced menstrual blood loss and achieved a difference compared with placebo in the proportion of patients with a PBAC score < 10 at weeks 6–12 (Fig. 3; *P* < .001 for all relugolix doses). As there was a difference in baseline total PBAC scores between the placebo and relugolix groups, we performed a subgroup analysis based on baseline total PBAC scores. This confirmed the result of the primary analysis. The subgroup analysis indicated that the proportion of patients with a total PBAC score < 10 at weeks 6–12 was higher in the relugolix 40-mg group than in any other treatment group, regardless of the baseline total PBAC score (Additional file 1: Table S3).

### Secondary endpoints

**Fig. 4:**
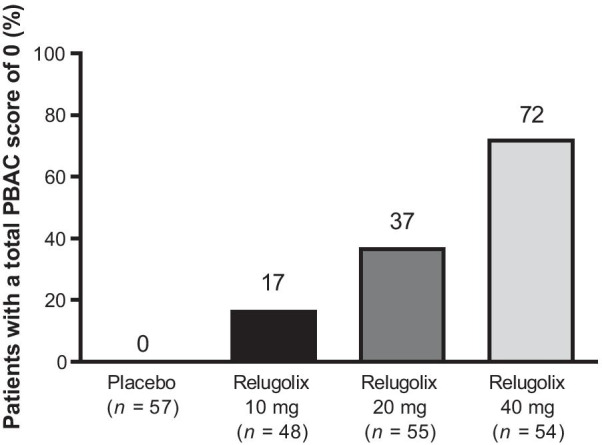
Secondary efficacy endpoints: proportion of patients with a total pictorial blood loss assessment chart (PBAC) score of 0 for weeks 6−12

The results of the analyses of PBAC scores < 10 for weeks 2–6 (Additional file 1: Fig. S1A) and weeks 2–12 (Additional file 1: Fig. S1B) were similar to the primary endpoint results. In addition, some patients achieved amenorrhea at weeks 6–12 of the study, with the highest proportion of these being in the relugolix 40-mg group (Fig. 4). No amenorrhea was seen in the placebo group. Similar results were observed for the proportion of patients who achieved amenorrhea at weeks 2–6 (Additional file 1: Fig. S1C) and 2–12 (Additional file 1: Fig. S1D).

Mean myoma and uterine volumes increased during the study in the placebo group, while the volumes in all relugolix groups decreased from week 2 and continued to decrease across weeks 4, 8, and 12, with the largest decrease in volumes observed in the relugolix 40-mg group (Additional file 1: Fig. S2). At week 12, uterine and myoma volumes were reduced by approximately 40% in the relugolix 40-mg group, compared with an increase of 10% in the placebo group.

Hemoglobin levels showed an increasing tendency following treatment in the relugolix 20- and 40-mg groups (Additional file 1: Fig. S3A). By week 12, the mean ± SD change from baseline in hemoglobin was 0.83 ± 1.17 g/dL and 0.93 ± 1.19 g/dL for the relugolix 20- and 40-mg groups, respectively, vs. 0.20 ± 1.00 g/dL for the placebo group. The blood concentrations of hemoglobin also tended to improve with relugolix treatment irrespective of the use of iron preparations (Additional file 1: Fig. S3B and Additional file 1: Fig. S3C).

**Fig. 5:**
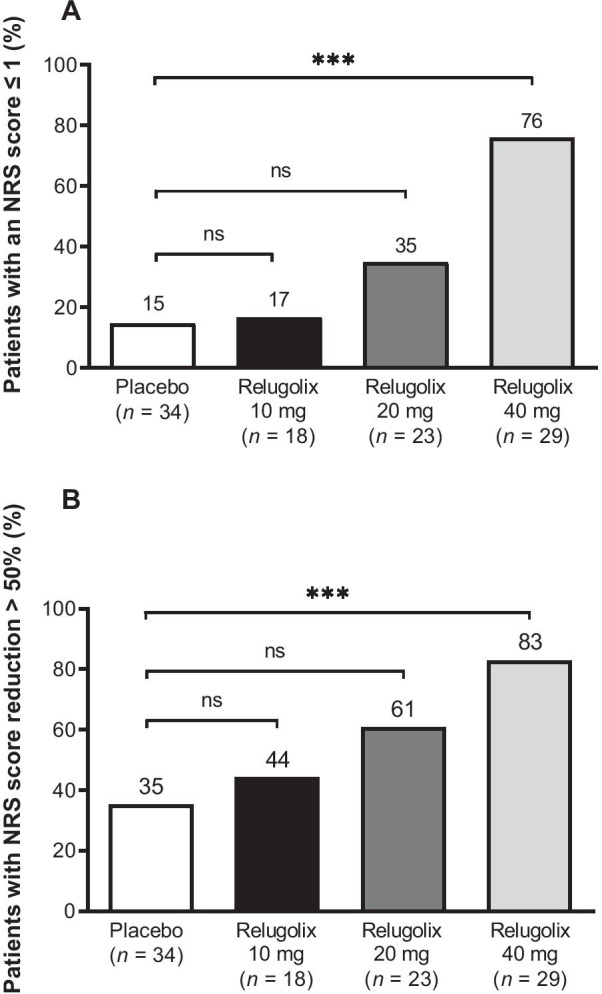
Proportion of patients with **A** maximum pain NRS score of 0 or 1 during the last 28 days before the last study dose, and **B** mean reduction in pain NRS score of > 50% from baseline to the last 28 days before the last study dose. Percentages are based on the number of patients with a maximum NRS score ≥ 4 at baseline. ***P < .001. *NRS* numerical rating scale, *ns* non-significant

Among women experiencing a pain severity of ≥ 4 on the NRS at baseline, significantly higher proportions of those in the relugolix 40-mg group achieved no pain or minimal pain during the last 28 days before the last dose of study medication, and reported a reduction in pain score of 50% or more (Fig. 5A and 5B, respectively; *P* < .001), compared with the placebo group. Treatment with relugolix also led to improvements in HRQL relative to placebo, with the lowest UFS-QOL symptom severity score and highest UFS-QOL HRQL total score observed in the relugolix 40-mg group (Additional file 1: Fig. S4A and Additional file 1: Fig. S4B).

### Pharmacodynamic effect-related endpoints

**Fig. 6:**
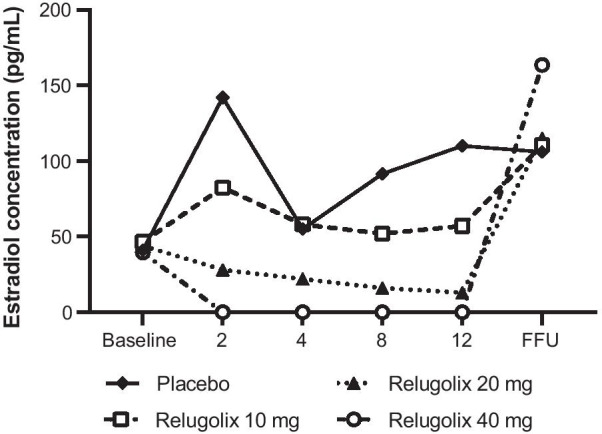
Median change in serum levels of estradiol. *FFU* final day of follow-up

During the treatment period, median serum estradiol concentration in the relugolix 40-mg group was reduced to < 10 pg/mL, the lower limit of quantification, at week 2, which was sustained until week 12 (Fig. 6), confirming complete suppression of ovarian function. Serum LH, FSH, and progesterone were also decreased with relugolix administration (Additional file 1: Fig. S5), with the lowest median LH and FSH levels seen in the 40-mg group (Additional file 1: Fig. S5A and Additional file 1: Fig. S5B).

### Safety

In this study, TEAEs were reported in 70.2% of patients in the placebo group, and 85.4–96.4% of patients in the relugolix groups (Table 2). In any relugolix groups, TEAEs with an incidence of ≥ 10% were nasopharyngitis, headache, metrorrhagia (irregular menstrual bleeding), menorrhagia (heavy menstrual bleeding), genital hemorrhage, hot flushes, and menstruation irregular. Of 35 patients reported with menorrhagia, 34 patients had been diagnosed with prolonged menstruation (menostaxis), which occurred mainly in the first menstrual cycle after treatment initiation, and all patients in the relugolix group had normal menstrual bleeding (with total PBAC scores < 100). Among these adverse events (AE), headache, metrorrhagia, menorrhagia, and hot flushes were reported in the relugolix 20- and 40-mg groups with similar incidence rates and at a ≥ 10% higher frequency relative to the placebo group. All TEAEs were mild or moderate in intensity except for one serious AE (SAE) of VIIth cranial nerve paralysis reported in the relugolix 40-mg group. Two other SAEs were reported: neurosensory deafness in one patient in the placebo group, and dysphonia in a 42-year-old patient in the relugolix 20-mg group. None of these SAEs were considered related to the study drug by the investigator. No deaths were reported during the study period. Two patients discontinued from the study because of TEAEs: one patient with decreased hemoglobin in the placebo group, and one patient with four events (tinnitus, decreased libido, depression, and hyperhidrosis) in the relugolix 20-mg group. One patient had a positive pregnancy test after receiving relugolix 10 mg for approximately 45 days. The study drug was discontinued and ultrasound confirmed that the patient was 7 weeks pregnant. The patient subsequently had an uneventful pregnancy and delivered a healthy infant at 39 weeks.

**Table 2:** Summary of treatment-emergent adverse events

	Relugolix					Placebo	
	10 mg (*n* = 48)	20 mg (*n* = 55)		40 mg (*n* = 54)		(*n* = 57)	
TEAEs, *n*	105	143		145		99	
Patients with any TEAEs	41 (85.4)	53 (96.4)		48 (88.9)		40 (70.2)	
Patients with drug-related TEAEs	33 (68.8)	50 (90.9)		44 (81.5)		23 (40.4)	
Intensity of TEAEs							
Mild	36 (75.0)	46 (83.6)		45 (83.3)		34 (59.6)	
Moderate	5 (10.4)	7 (12.7)		2 (3.7)		6 (10.5)	
Severe	0 (0.0)	0 (0.0)		1 (1.9)		0 (0.0)	
TEAEs leading to study drug discontinuation	0 (0.0)	1 (1.8)		0 (0.0)		1 (1.8)	
Serious TEAEs (%)	0 (0.0)	1 (1.8)		1 (1.9)		1 (1.8)	
TEAEs occurring in ≥ 10% of patients in any group							
Nasopharyngitis	9 (18.8)	4 (7.3)		7 (13.0)		16 (28.1)	
Hot flushes	2 (4.2)	16 (29.1)		21 (38.9)		2 (3.5)	
Irregular uterine bleeding	13 (27.1)	17 (30.9)		15 (27.8)		10 (17.5)	
Heavy menstrual bleeding	6 (12.5)	13 (23.6)		12 (22.2)		4 (7.0)	
Headache	1 (2.1)	8 (14.5)		7 (13.0)		1 (1.8)	
Genital hemorrhage	2 (4.2)	6 (10.9)		6 (11.1)		2 (3.5)	
Menstruation irregular	12 (25.0)	8 (14.5)		3 (5.6)		0 (0.0)	

Data are *n* (%) unless stated otherwise

*TEAEs* treatment-emergent adverse events

A decrease in BMD was observed from baseline to week 12, with mean ± SD percentage changes of − 0.75 ± 2.35%, − 1.93 ± 2.26%, and − 2.27 ± 2.21% in the relugolix 10-, 20-, and 40-mg groups, respectively, compared with − 0.24 ± 2.22% in the placebo group.

The menstrual cycle resumed in 98% of the 214 patients who were included in the safety analysis, but not in five patients because of pregnancy (one patient in the relugolix 10-mg group) or the initiation of hormonal therapy for treatment of uterine leiomyoma and heavy menstrual bleeding before the resumption of the menstrual cycle in four patients (one patient in the 20-mg group and three in the 40-mg group). The median (Q1, Q3) duration from the last dose of study drug to the resumption of menstrual cycles was 22.0 (10.0, 27.0), 28.0 (23.0, 34.0), and 37.0 (34.0, 40.0) days in the relugolix 10-, 20-, and 40-mg groups, respectively, compared with 19.0 (14.0, 24.0) days in the placebo group.

No clinically significant changes in laboratory values, vital signs, weight, or ECG findings were observed for patients in any treatment groups during the study.

## Discussion

The present study was the first to demonstrate that relugolix at doses of 10, 20, and 40 mg yielded significant reductions in menstrual blood volume associated with uterine leiomyoma. These findings are consistent with and extend those of the Phase 3 relugolix studies [12, 13]. The primary endpoint (a total PBAC score of < 10 at weeks 6–12) was achieved by 20.8–83.3% of patients receiving relugolix and 0% of patients receiving placebo. The greatest benefit in patients with heavy menstrual bleeding due to uterine leiomyoma was achieved with the 40-mg dose of relugolix. This dose–response was evident within 2–6 weeks, supporting the rapid onset of the relugolix effect. These results were clinically significant, given that heavy menstrual bleeding is a serious health concern in patients with leiomyoma [6].

Similarly, a benefit of relugolix was observed for all secondary endpoints, including pain, which is a highly patient-relevant symptom linked to HRQL impairment due to uterine leiomyomas [6]. Although the patients enrolled in the study did not report large improvements in UFS-QOL scores, this was likely due to the enrolled patients experiencing less severe HRQL impairment at baseline (based on the symptom severity and total HRQL scores). The greatest improvements in UFS-QOL scores were observed for those patients receiving relugolix 40 mg. This is similar to the findings from a large phase 3 study of relugolix 40 mg in Japanese women with heavy menstrual bleeding that reported improved HRQL scores at week 12, which were sustained through week 24 [13]. A numerical increase in hemoglobin levels observed in patients receiving relugolix may mitigate iron-deficiency anemia, which is a potentially life-threatening aspect of the morbidity associated with leiomyomas [3, 4].

Large myomas and an enlarged uterus may result in “bulk” symptoms, such as back, leg, or pelvic pressure, and bowel and bladder obstruction [1, 3, 8]. All doses of relugolix reduced myoma and uterine volumes, with the greatest decrease in the 40-mg group, contrasting with the increased volumes seen in the placebo group. Bulk reductions reflect the dependence of myoma growth on estrogen and progesterone [18] and the mechanism of action of relugolix; by week 2, median estrogen concentration in the relugolix 40-mg group was undetectable, reflecting complete suppression of ovarian function [19].

Relugolix was generally well tolerated. Although two SAEs were reported in the relugolix groups, neither was related to the study drug in the judgment of the investigator. Common TEAEs reported more frequently with relugolix than placebo were consistent with the hormonal changes due to relugolix monotherapy and the subsequent hypoestrogenic state. Importantly, heavy menstrual bleeding was recorded as menostaxis (excessively long menstruation), which mainly occurred in the first menstrual cycle after treatment initiation. This is considered to be a well-known phenomenon in patients treated with products that affect their reproductive hormonal status, such as oral contraceptives [20, 21]. The total score of PBAC during the occurrence of such adverse reactions was less than the menstrual blood loss at baseline. The reduction in BMD observed in the relugolix groups was likely due to estrogen suppression. The 2.27% mean decrease in BMD of lumbar spine from baseline to week 12 observed in the 40-mg group in our study is in line with the 2.7% mean decrease following 3 months of treatment with 3.75 mg of the GnRH agonist, leuprolide acetate, in another uterine leiomyoma study [22]. GnRH agonists are commonly used for short-term uterine leiomyoma treatment before surgery but, unlike relugolix, require parenteral administration, may cause temporary worsening of symptoms, and typically take 3–4 weeks before a therapeutic effect emerges [1, 3, 8, 9].

Although BMD loss rules out extended monotherapy with relugolix, it may be a suitable option for short-term presurgical treatment of uterine leiomyomas. In addition, long-term treatment may be feasible based on the estrogen threshold hypothesis [23]. The concentrations of estrogen necessary to stimulate leiomyomas are higher than those required to maintain BMD, thus there may be a therapeutic window in which a low amount of estradiol may be administered in combination with relugolix to prevent BMD loss, while still achieving benefit for the symptoms of leiomyomas [24]. Indeed, the efficacy and safety of relugolix 40 mg once daily co-administered with low-dose estradiol (1 mg) and norethindrone acetate (0.5 mg) have been assessed in the LIBERTY 1 and LIBERTY 2 phase 3 trials (ClinicalTrials.gov Identifiers NCT03049735 and NCT03103087); in these trials, BMD loss was similar with relugolix combination therapy and placebo [25]. Although it is difficult to compare monotherapy directly with combination therapy, the overall efficacy and safety outcomes of the LIBERTY trials were similar to the present study.

Strengths of this study include the prospective, multicenter, randomized, double-blind, and placebo-controlled study design. Furthermore, the findings of this study are consistent with those of the Japanese phase 3 studies [12, 13], lending further support to the reported benefits of relugolix in treating uterine leiomyomas. This study has several limitations. First, the assessment period was only 12 weeks, the common duration for preoperative treatment but insufficient for evaluating long-term treatment. Second, only Japanese patients were included in the study. Finally, the patient population may not permit adequate assessment of uncommon AEs.

In summary, relugolix may provide a new therapeutic option for treatment of uterine leiomyoma symptoms in premenopausal women, with substantial benefit achieved at a 40-mg dose. Ongoing studies will determine the efficacy and safety of long-term treatment.

## Supplementary Information

**Additional file 1.** Study assessments, inclusion/exclusion criteria, results of PBAC subgroup analysis, and secondary efficacy endpoints. 

## Data Availability

The datasets generated and/or analyzed during the current study are available by request from vivli.org. The datasets, including the redacted study protocol, redacted statistical analysis plan, and individual participants data supporting the results reported in this article, will be made available within three months from initial request, to researchers who provide a methodologically sound proposal. The data will be provided after its de-identification, in compliance with applicable privacy laws, data protection and requirements for consent and anonymization.

## Abbreviations

AE: Adverse events
BMD: Bone mineral density
BMI: Body mass index
CI: Confidence interval
ECG: 12-lead electrocardiogram
FFU: Final day of follow-up
FSH: Follicle-stimulating hormone
GnRH: Gonadotropin-releasing hormone
HRQL: Health-related quality of life
LH: Luteinizing hormone
NRS: Numerical rating scale
PBAC: Pictorial blood loss assessment chart
SAE: Serious adverse events
SD: Standard deviation
TEAEs: Treatment-emergent adverse events
UFS-QOL: Uterine Fibroid Symptom and HRQL Questionnaire.
