# Supplementary material for: Relugolix for oral treatment of uterine leiomyomas: a dose-finding, randomized, controlled trial
Source: BMC Womens Health. 2021 Oct 28;21:375. doi: 10.1186/s12905-021-01475-2 (PMC8555132; doi:10.1186/s12905-021-01475-2)

Supplementary Materials

Table S1 Study assessments

	Pretreatment		Treatment					
	Screening	Placebo run-in (3–6 wks)	Day 1	Week 2	Week 4	Week 8	Week 12	Follow-up (4 wks)
Visit	1	2	3	4	5	6	7	8
Demography, baseline characteristics	X							
Patient diary (PBAC and NRS)	←	—	—	—	—	—	→	
Transvaginal ultrasound scan	X		X	X	X	X	X	
Hemoglobin	X	X	X		X	X	X	X
UFS-QOL			X		X	X	X	
Pharmacodynamic measures[a]			X	X	X	X	X	X
Menstruation recovery								X
AEs	X	X	X	X	X	X	X	X
Vital signs, weight	X	X	X		X	X	X	X
Physical examination	X	X	X	X	X	X	X	X
Clinical laboratory tests[b]	X	X	X		X	X	X	X
12-lead ECG	X		X		X	X	X	X
BMD		X					X	
Pregnancy test[c]	X				X	X	X	X
Concomitant medications	X	X	X	X	X	X	X	X

Abbreviations: AE adverse event, *BMD* bone mineral density, *ECG* electrocardiogram, *NRS* numerical rating scale, *PBAC* pictorial blood loss assessment chart, *UFS-QOL* Uterine Fibroid Symptom Health-Related Quality of Life Questionnaire

[a]Blood estradiol, luteinizing hormone, follicle-stimulating hormone, progesterone

[b]Serum chemistry, hematology, and urinalysis

[c]Not conducted if the subject was menstruating at the visit

Table S2 Inclusion/exclusion criteria

Inclusion criteria	Exclusion criteria
≥ 20 years of age	Blood disorders[c]
Diagnosis of uterine leiomyoma[a], with ≥ 1 measurable noncalcified myoma[b]	Lower abdominal pain due to irritable bowel syndrome or severe interstitial cystitis
Regular menstrual cycle (25–38 days)	Thyroid dysfunction
Heavy menstrual bleeding in one menstrual cycle, total PBAC score ≥ 120	Pelvic inflammatory disease
	Positive Pap smear test result
	History of hysterectomy or bilateral oophorectomy[d]

Abbreviation: PBAC pictorial blood loss assessment chart
[a]Confirmed by transvaginal ultrasound scan, magnetic resonance imaging, computed tomography scan, or laparoscopy
[b]Widest diameter ≥ 3 cm
[c]Other than iron-deficiency anemia
[d]Or history of other medical conditions that could interfere with participation in the study

Table S3 Results of the subgroup analysis of total PBAC score

Baseline PBAC score subgroup	Treatment	*n*	Proportion of patients with total PBAC score < 10 from weeks 6–12 (%)
120 ≤ PBAC < 200	Placebo	20	0
	Relugolix 10 mg	23	30.4
	Relugolix 20 mg	25	48.0
	Relugolix 40 mg	26	80.8
200 ≤ PBAC < 500	Placebo	27	0
	Relugolix 10 mg	20	15.0
	Relugolix 20 mg	21	38.1
	Relugolix 40 mg	26	84.6
500 ≤ PBAC < maximum	Placebo	10	0
	Relugolix 10 mg	5	0
	Relugolix 20 mg	8	37.5
	Relugolix 40 mg	2	100

Abbreviation: PBAC pictorial blood loss assessment chart

Supplementary Fig. S1 Secondary efficacy endpoints: **A.** Proportion of patients with a total pictorial blood loss assessment chart (PBAC) score of <10 for weeks 2-6. **B.** Proportion of patients with a PBAC score < 10 for weeks 2-12. **C.** Proportion of patients with a total PBAC score of 0 for weeks 2-6. **D.** Proportion of patients with a total PBAC score of 0 for weeks 2-12.

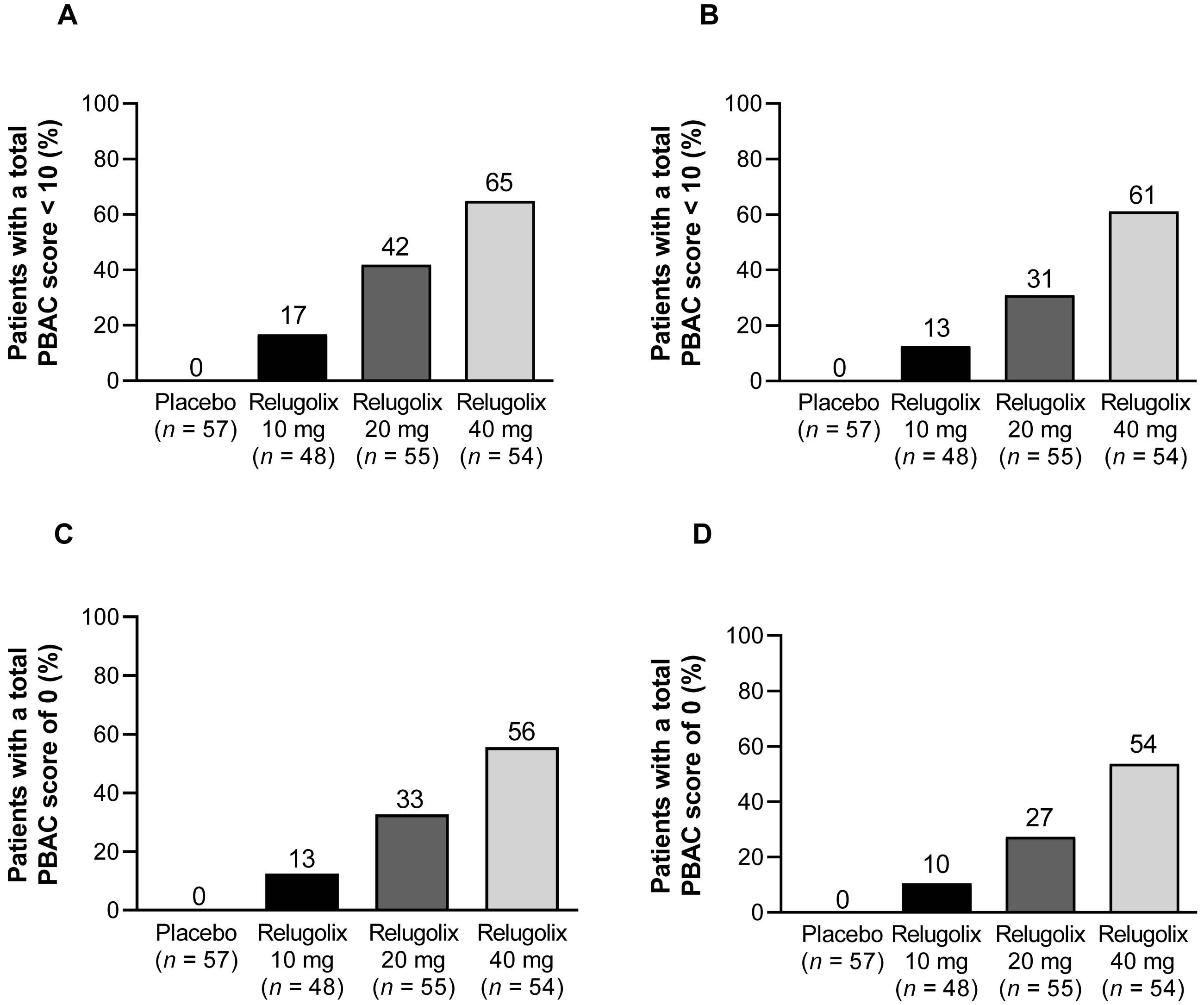

Supplementary Fig. S2. Secondary efficacy endpoints: **A.** Mean percent change from baseline in myoma volume. **B.** Mean percent change from baseline in uterine volume.

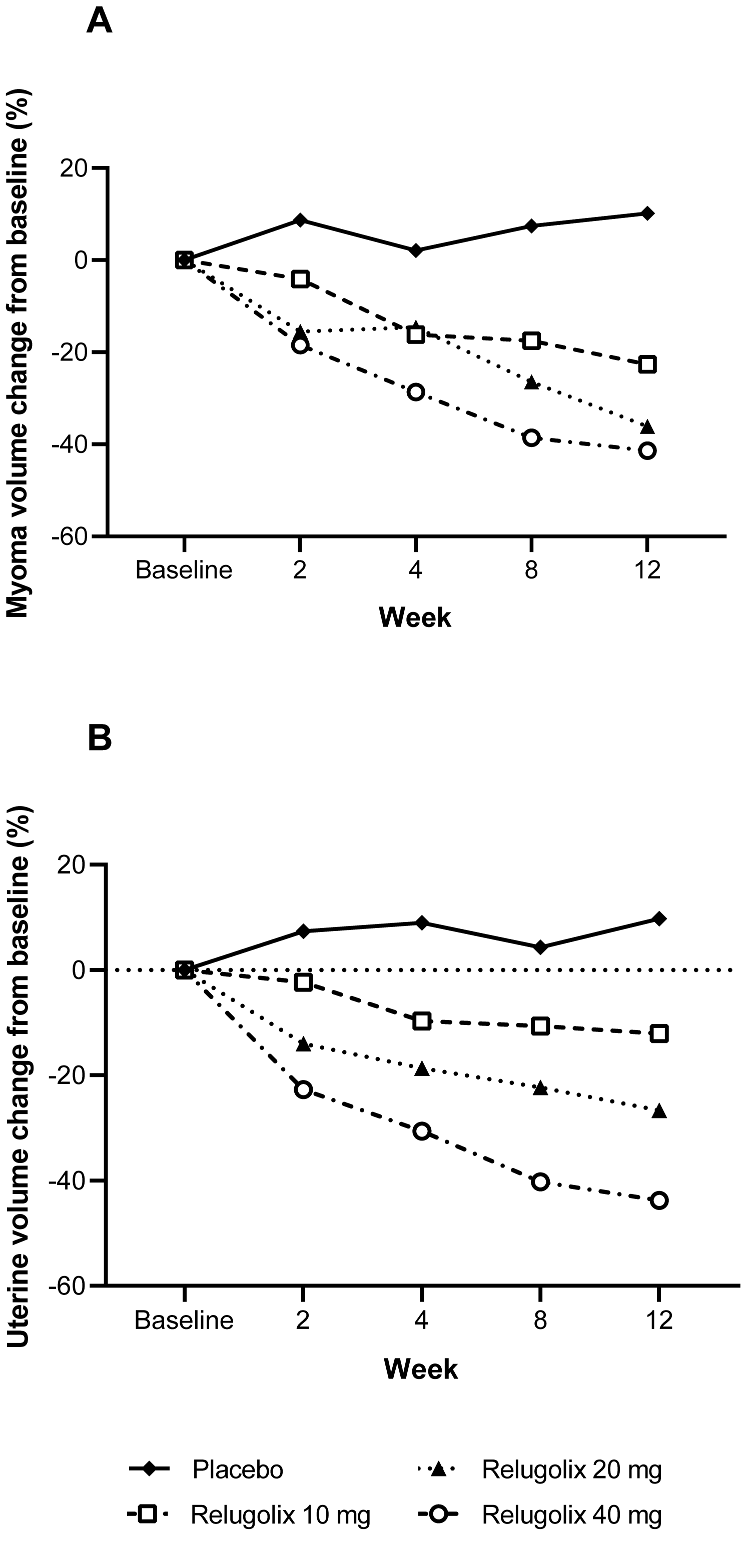

Supplementary Fig. S3. Secondary efficacy endpoint: Change from baseline in serum level of hemoglobin.

A. Overall. **B**. Without oral iron. **C**. With oral iron.

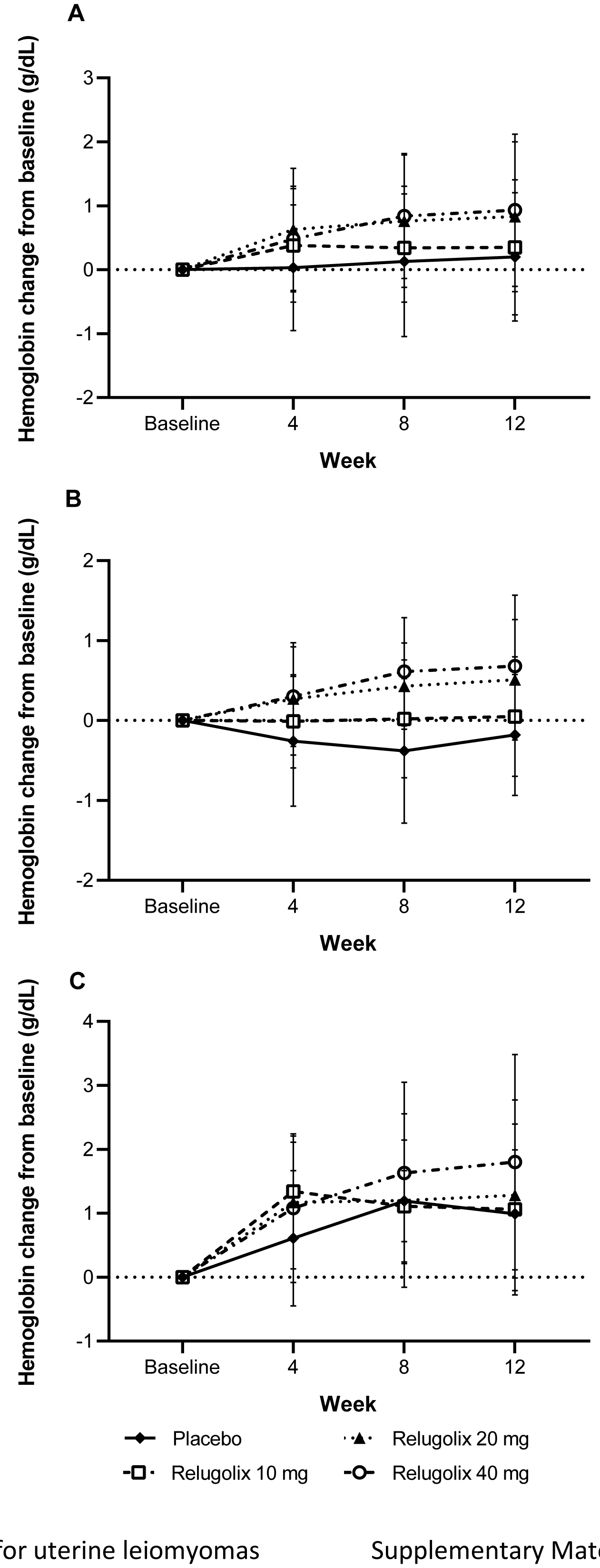

Supplementary Fig. S4. Secondary efficacy endpoint: Mean change from baseline in UFS-QOL score. **A**. Symptom severity score, **B**. HRQL total score. HRQL, health-related quality of life.

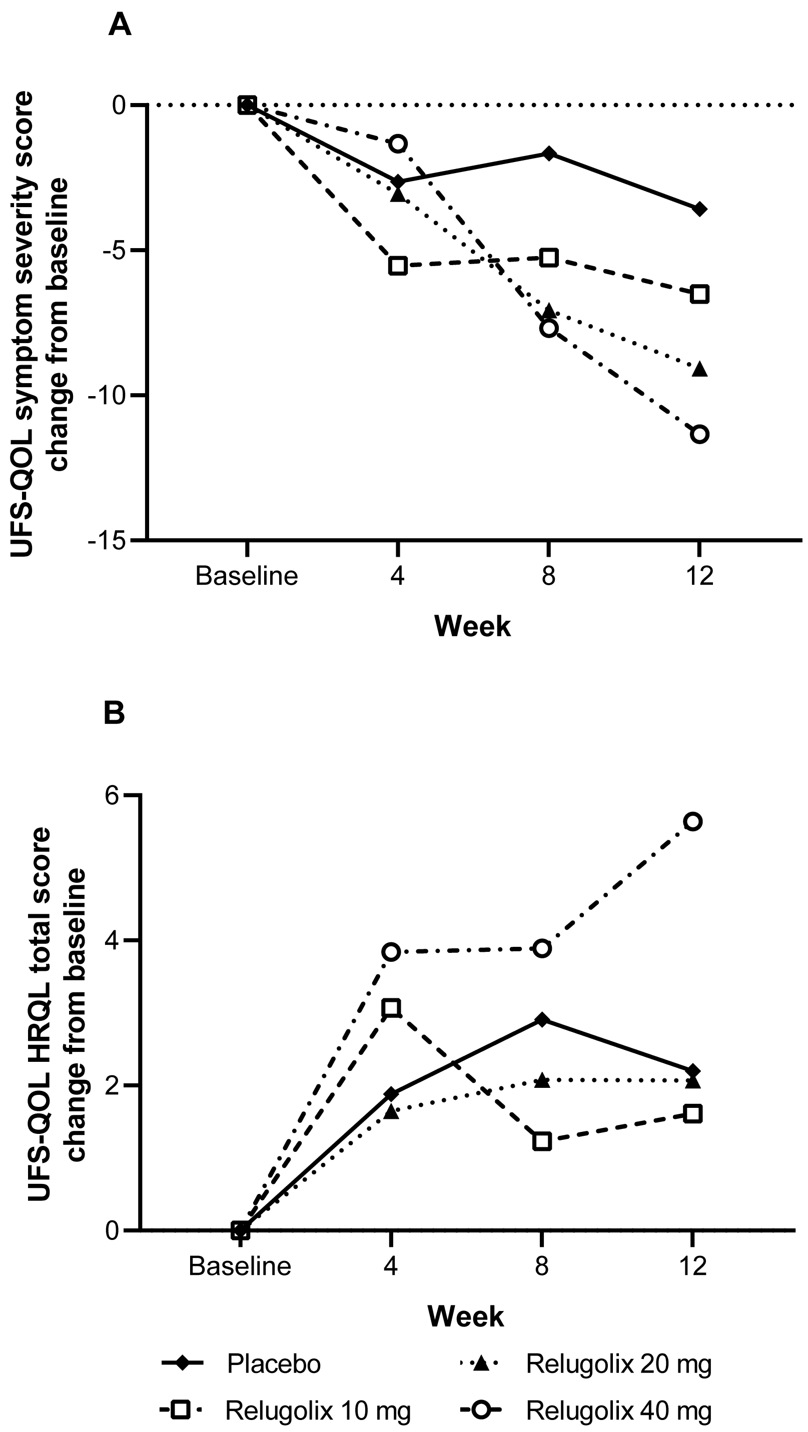

Supplementary Fig. S5. Change in serum levels of (**A**) LH, (**B**) FSH, and (**C**) progesterone across the study. Data are the medians. LH, luteinizing hormone; FSH, follicle-stimulating hormone; FFU, the final day of follow-up.

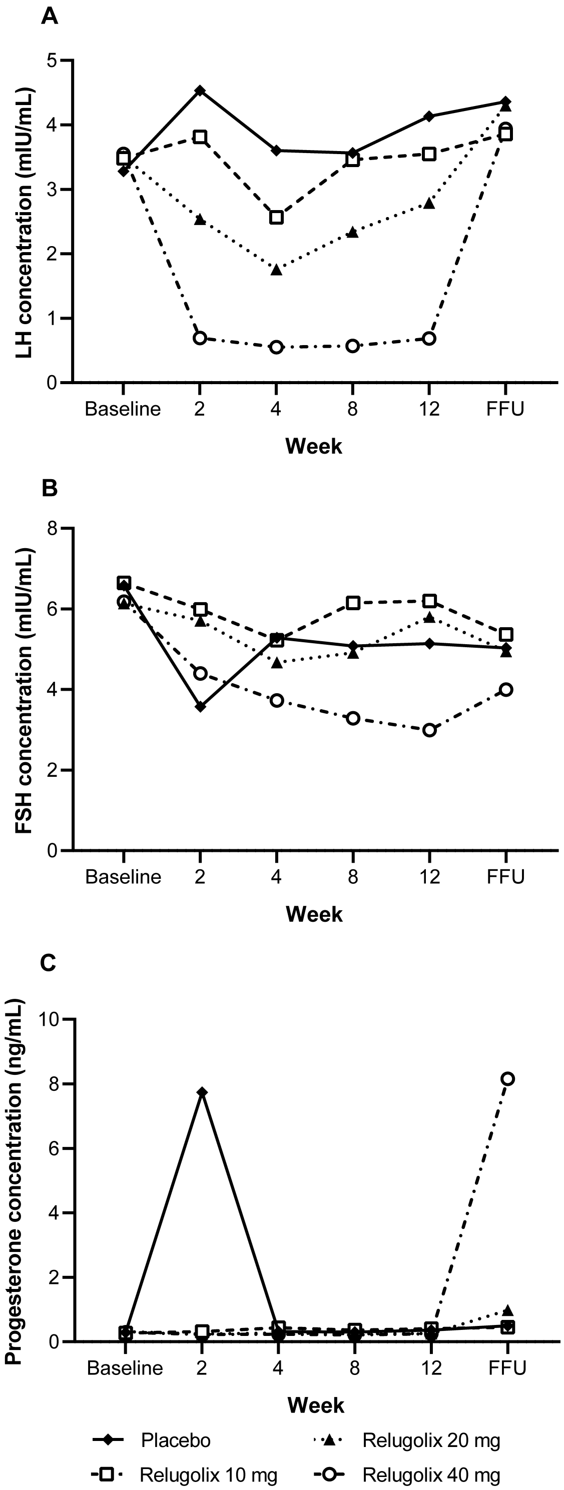

Supplement: Supplementary file 1 — Additional file 1. Study assessments, inclusion/exclusion criteria, results of PBAC subgroup analysis, and secondary efficacy endpoints. [file 12905_2021_1475_MOESM1_ESM.pdf]
